# Supplementary material for: Bacillamide D produced by Bacillus cereus from the mouse intestinal bacterial collection (miBC) is a potent cytotoxin in vitro
Source: Commun Biol. 2024 May 28;7:655. doi: 10.1038/s42003-024-06208-3 (PMC11133360; doi:10.1038/s42003-024-06208-3)
Supplement: Supplementary file 2 — Supplementary Information [file 42003_2024_6208_MOESM2_ESM.pdf]

Bacillamide D produced by Bacillus cereus from the mouse intestinal bacterial collection (miBC) is a potent cytotoxin in vitro

Maximilian Hohmann,[1] Valentina Brunner,[2,3,4] Widya Johannes,[5] Dominik Schum,[6] Laura M. Carroll,[7] Tianzhe Liu,[1] Daisuke Sasaki,[5,8] Johanna Bosch,[9] Thomas Clavel,[9] Stephan A. Sieber,[6] Georg Zeller,[7] Markus Tschurtschenthaler,[2,3,4*] Klaus-Peter Janßen,[5*] Tobias A. M. Gulder[1,10*]

[1] Chair of Technical Biochemistry, Technical University of Dresden, Bergstraße 66, 01069 Dresden, Germany.

[2] Chair of Translational Cancer Research and Institute of Experimental Cancer Therapy, Klinikum rechts der Isar, School of Medicine, Technical University of Munich, 81675 Munich, Germany.

[3] Center for Translational Cancer Research (TranslaTUM), Klinikum rechts der Isar, School of Medicine, Technical University of Munich, 81675 Munich, Germany.

[4] Division of Translational Cancer Research German Cancer Research Center (DKFZ) and German Cancer Consortium (DKTK), 69120 Heidelberg, Germany.

[5] Department of Surgery, Klinikum rechts der Isar, School of Medicine, Technical University of Munich, 81675 Munich, Germany.

[6] Department of Bioscience, Center for Functional Protein Assemblies, Technical University of Munich, 85748 Garching bei München, Germany.

[7] Structural and Computational Biology Unit, European Molecular Biology Laboratory, 61997 Heidelberg, Germany.

[8] Research and Development Headquarters, Nitto Boseki Co., Ltd., 102-8489 Tokyo, Japan.

[9] Functional Microbiome Research Group, Institute of Medical Microbiology, University Hospital of RWTH Aachen, 52074 Aachen, Germany.

[10] Helmholtz Institute for Pharmaceutical Research Saarland (HIPS), Department of Natural Product Biotechnology, Helmholtz Centre for Infection Research (HZI) and Department of Pharmacy at Saarland University, Campus E8.1, 66123 Saarbrücken, Germany.

SUPPORTING INFORMATION

Table of Contents

Supplementary Tables 2

Supplementary Figures 5

Supplementary Data 12

NMR-Data 13

Supplementary References 16

Supplementary Tables

Table S1. Bacillamide D did not show antibacterial activity against the tested panel of bacterial pathogens.

**strain**	**medium**	**MIC [µM]**
Staphylococcus aureus USA300 Lac (JE2)	LB (Sigma-Aldrich) + 0.1% K_2HPO_4	> 1 mM
Klebsiella pneumoniae DSM 30104	BHI (Roth)	> 1 mM
Acinetobacter baumanii DSM 30007	BHI (Roth)	> 1 mM
Pseudomonas aeruginosa PAO1	LB (Sigma-Aldrich)	> 1 mM
Enterobacter cloacae subsp. cloacae DSM 30054	BHI (Roth)	> 1 mM
Escherichia coli 536	LB (Sigma-Aldrich)	> 1 mM
Listeria monocytogenes EGD-e	BHI (Roth)	> 1 mM
Enterococcus faecalis V583	BHI (Roth)	> 1 mM

Table S2. Bacterial strains, plasmids and organoid lines used in this study.

Strains	Description	Reference/Source
E. coli DH5α	Host strain for cloning	NEB
E. coli BAP1	Heterologous expression strain	Pfeifer et al., 2001[1]
Bacillus cereus DSM 28590	Bacterial strain from miBC (www.dsmz.de/miBC), harbouring the *bac* BGC	Lagkouvardos et al., 2016[2]
**Plasmids**	**Description**	**Reference/Source**
pET28b-ptetO::*gfp* (6552 bp)	Tetracycline inducible expression plasmid ColE1, Kan^R, *gfp* reporter gene downstream of promotor	Duell et al., 2019[3]
pET28b-ptetO::*bac*::*gfp* (16052 bp)	pET28b-ptetO-*gfp*v2 with bac-cluster cloned between ptetO and *gfp*	This study
**Organoid Lines**	**Description**	**Reference/Source**
B6-2 Duo	Healthy intestinal duodenum organoid line	This study
B6-2 Colon	Healthy intestinal colon organoid line	This study
MT577 T1-2	Intestinal duodenum tumor organoid line	Felchle et al., 2023[4]
MT1473 T4	Intestinal colon tumor organoid line	This study

Table S3. List of oligonucleotides used for cloning and screening purposes.

Name	Sequence (5' → 3')	Description
C-GFP_for_1	CATGGTTAGCAAAGGTGAAG	Amplification of pET28b-ptetO-gfp backbone
Spec-ptet-R	GGTCGATCCTCTTCTCTATC	
gib_ptet_G72C12_I_F	AGTGATAGAGAAGAGGATCGACCATGAGCGAGAAAATTTATTACTGGTC	Amplification of first part of bacillamide cluster with homology arms
gib_ptet_G72C12_I_R	TGTCATTTATAATTTGTAATCACAAATTGTTTGTATTGG	
gib_ptet_G72C12_II_F	CAAACAATTTGTGATTACAAATTATAAATGACAAAAAAATTACAGTTATCAGCC	Amplification of second part of bacillamide cluster with homology arms and **StuI** restriction site in reverse primer
gib_ptet_G72C12_II_StuI_R	GTTCTTCACCTTTGCTAACCATG**AGGCCT**CCGCCTAATATATCAAGGTTGAG	
Screen_ptetF2	TCCGACCTCATTAAGCAGC	Colony screening
Screen-G72C12-dehydro-Rev	TGACGACAAGACCTACGTTCC	Colony screening
Seq-G72C12-jct-Fwd	TTTGCTATCACGGATTACGAAG	Colony screening
Seq-G72C12-jct-Rev	AAATGAGGATGATCCACATGAG	Colony screening
Screen-G72C12-ami-Fwd	ACCTGCCCAATTAGGAATTGTTAC	Colony screening
Screen_GFP_R	TTACCGTTGGTCGCATCACC	Colony screening

Supplementary Figures

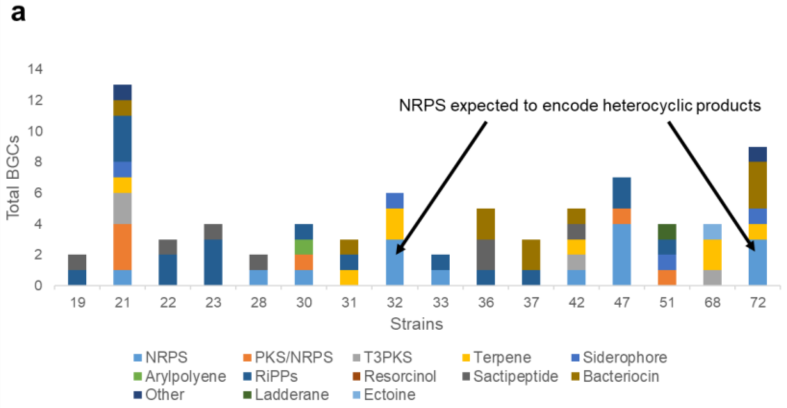

b

Strain No	Strain Name	DSM
19	Lachnospiraceae sp.	29356
21	Paenibacillus lactis	28626
22	Lachnospiraceae sp.	28559
23	Enterococcus hirae	28619
28	Clostridium boltae	29485
30	Escherichia coli	28618
31	Enterococcus hirae	32036
32	Staphylococcus xylosis	28566
33	Enterococcus gallinarum	100110
36	Clostridium bifermentans	29423
37	Lactobacillus taiwanesis	100220
42	Cellulosimicrobium cellulans	29131
47	Blautia coccoides	28737
51	Proteus mirabilis	28629
68	Oceanobacillus caeni	28620
72	Bacillus cereus	28590

Figure S1: **Results of genome mining of miBC strains**. **a** Selection of most promising strains in terms of biosynthetic capability; BGCs identified by AntiSMASH are grouped by family. Clusters encoded by strains 32 and 72 were predicted to encode heterocyclic natural products, either by presence of heterocyclization or thioreductase domains. **b** List of strains from A with strain names and DSM numbers.

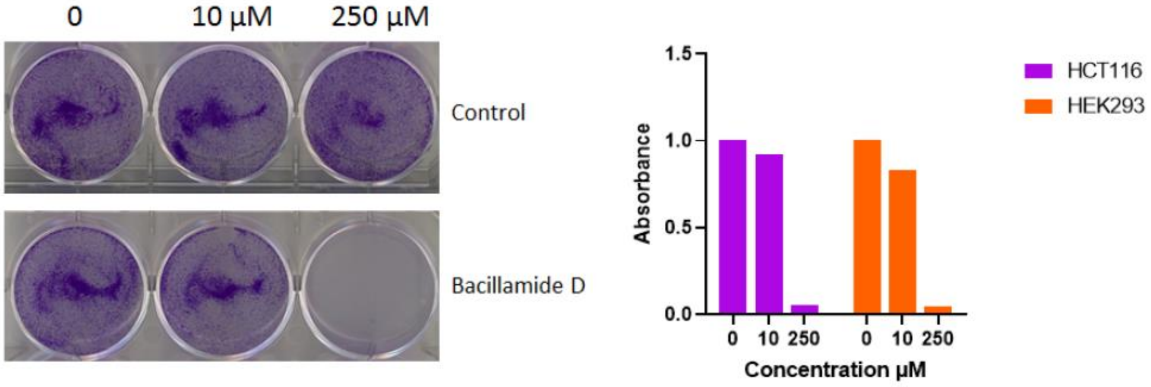

Figure S2. Crystal-violet staining assay initially shows growth inhibiting activity of bacillamide D at 250 μM concentration.

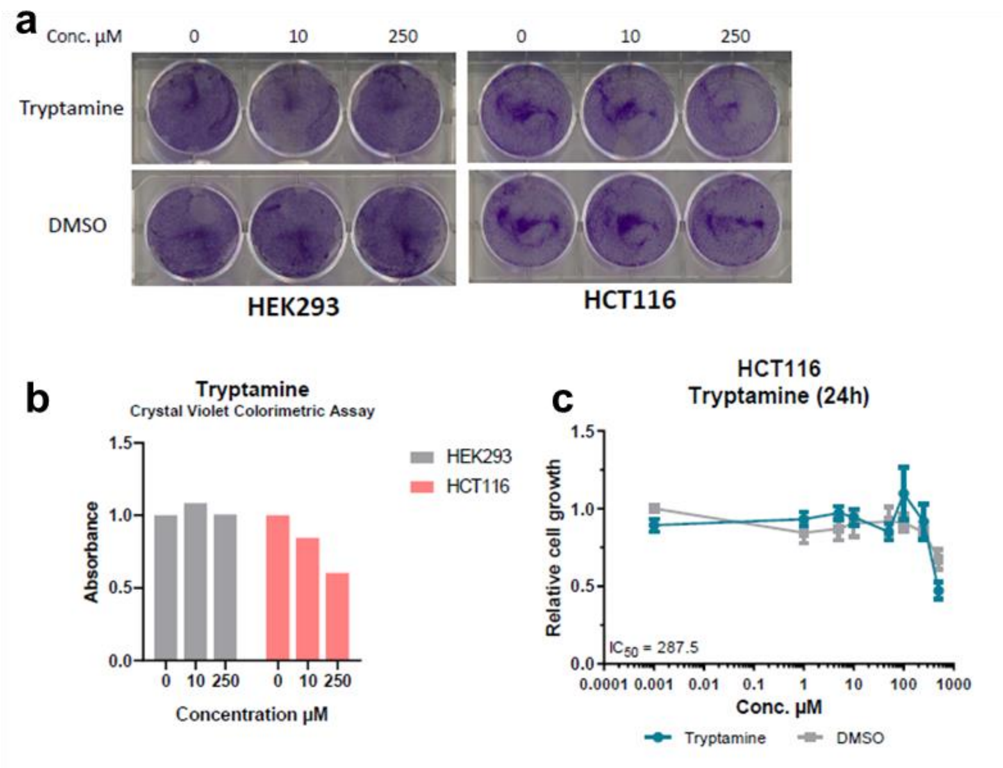

Figure S3. Weak growth-inhibiting activity of tryptamine on HEK293 and HCT116 cells. **a** and **b** Crystal-violet staining assays; **c** XTT metabolic growth assay with HCT116 cells establishes an IC_{50}-value of 289.5 µM (n=6).

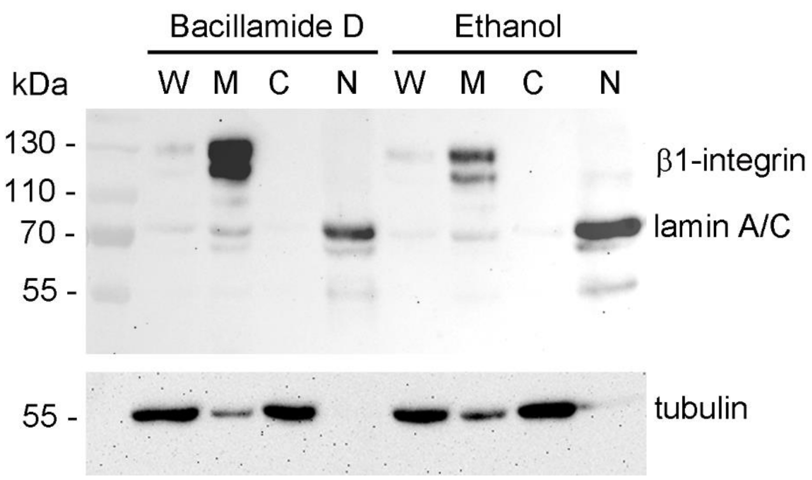

Figure S4. Western blot of subcellular fraction samples after 24h treatment for the presence of marker proteins: L – protein ladder; W – whole cell fraction; M – membrane fractions with β1-integrin bands; C – cytosolic fraction with tubulin band; N – nuclei with lamin a/c bands.

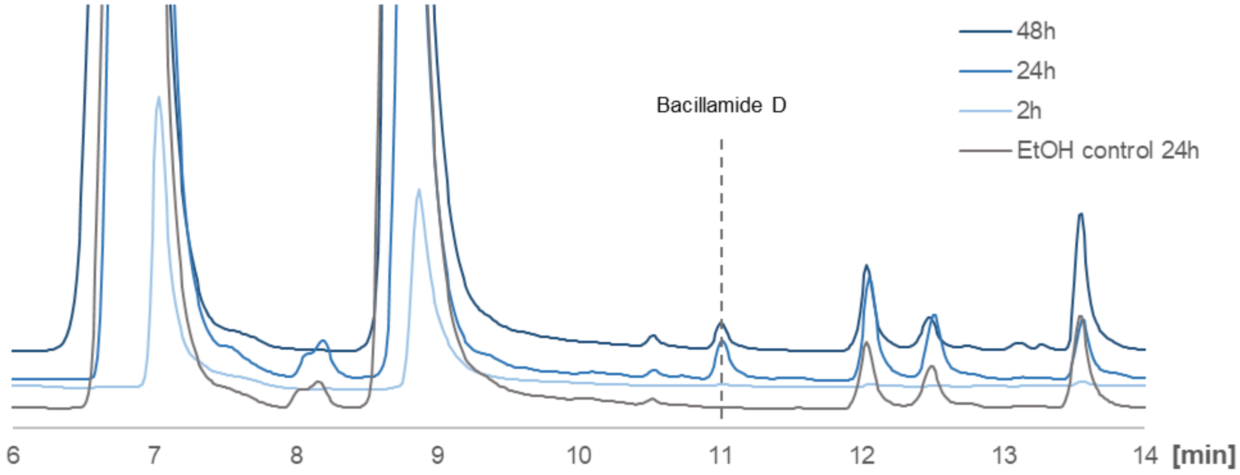

Figure S5. Uptake of bacillamide D into cytosolic fractions of HCT116 cells at different time points, compared to negative EtOH control.

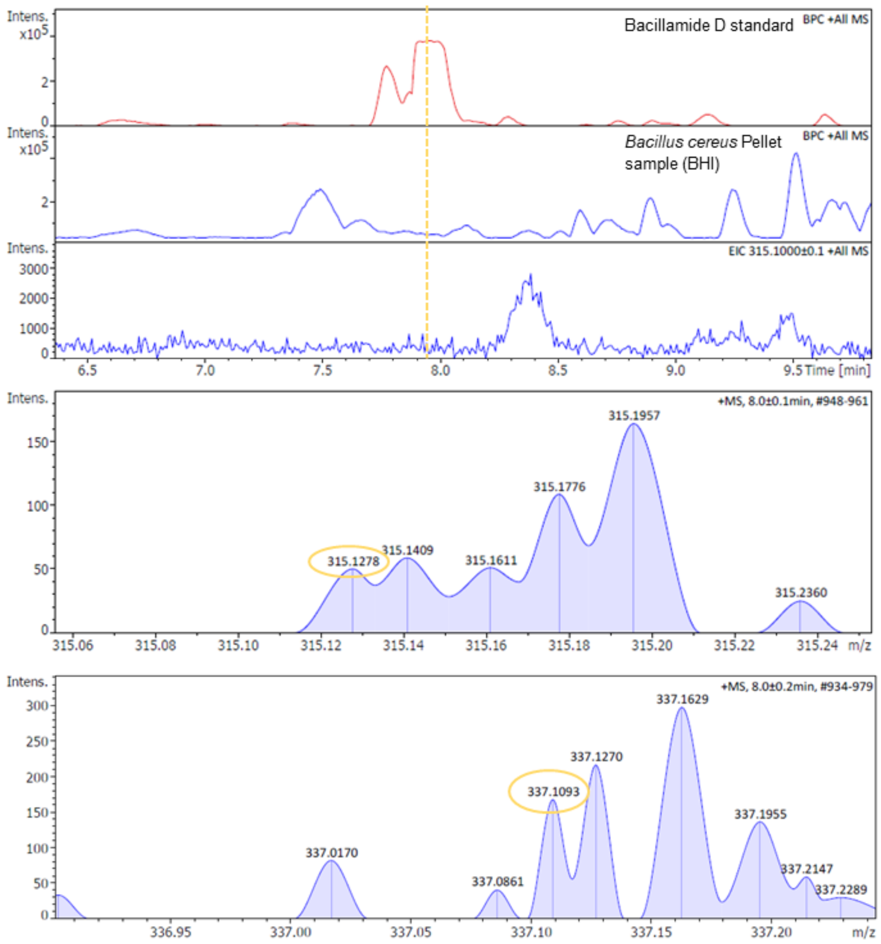

Figure S6. Detection of bacillamide D in pellet sample of *Bacillus cereus*. The strain was grown over 24 h in BHI and the pellet extracted with methanol as described previously. HR-MS (ESI+): m/z = 315.1278 $[M+H]^+$, calc.: 315.1274; m/z = 337.1093 $[M+Na]^+$, calc.: 337.1094. The retention time of the identified signals matched a bacillamide D reference sample.

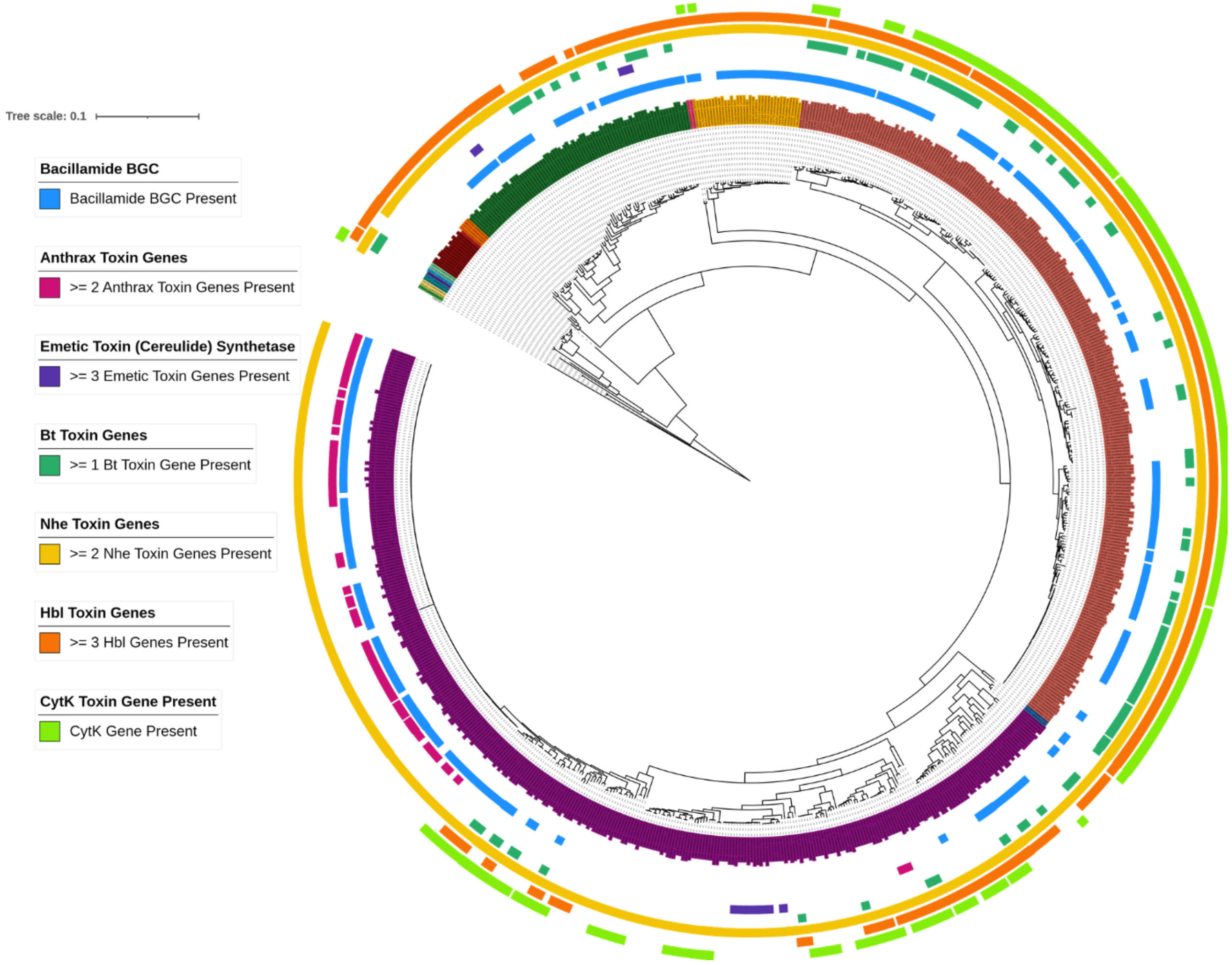

Figure S7. Taxonomic spread of the *bac* BGC in *Bacillus cereus* group strains. All *Bacillus cereus* group genomes in the proGenomes v2 database were downloaded.[5] The quality of all genomes was evaluated using (i) QUAST v5.0.2 (using default settings, plus "--min-contig" set to "1"[6] and (ii) the "lineage_wf" command in CheckM v1.1.3 (default settings)[7];

genomes with CheckM completeness ≥95 and contamination ≤5 were used in subsequent steps (see Data S1 for proGenomes v2 accession numbers). Each genome was queried for the presence of the *bac* BGC by querying for the presence of the NRPS protein among all genome proteins using blastp v2.12.0 (identity>90%, alignment length >90% query length). Prokka v1.14.6[8] was used to annotate each genome (using default settings and the "Bacteria" database), and the resulting annotated open reading frames were supplied as input to Panaroo v1.2.7.[9] Panaroo was used to construct a core genome alignment, using the following parameters: "--clean-mode strict -a core --aligner mafft --core_threshold 0.95 -f 0.5". The resulting core gene alignment was supplied to IQ-TREE v1.5.4,[10] and IQ-TREE was used to construct a maximum likelihood phylogeny using the general time-reversible nucleotide substitution model and one thousand replicates of the ultrafast bootstrap approximation.[11] The resulting phylogeny was displayed in iTOL v6,[12] with (from interior to exterior): (i) tip labels colored by genomospecies assigned using BTyper3 v3.3.3 (default settings)[13]; (ii) presence and absence of *bac* ("Bacillamide BGC", blue tiles); presence and absence of (iii) two or more anthrax toxin genes ("Anthrax Toxin Genes", pink tiles), (iv) three or more emetic toxin (cereulide) synthetase genes ("Emetic Toxin [Cereulide] Synthetase", purple tiles), (v) one or more Bt insecticidal toxin genes ("Bt Toxin Genes", dark green tiles), (vi) two or more non-hemolytic enterotoxin (Nhe)-encoding genes ("Nhe Toxin Genes", yellow tiles), (vii) three or more hemolysin BL (Hbl) toxin-enoding genes ("Hbl Toxin Genes", orange tiles), and (viii) CytK toxin-encoding *cytK* ("CytK Toxin Gene Present", light green tiles), detected using BTyper3 v3.3.3 (default settings)[14]. The tree is rooted using an outgroup genome (i.e., *B. panaciterrae*, NCBI Assembly accession GCF_000430785.1), and branch lengths are reported in substitutions per site.

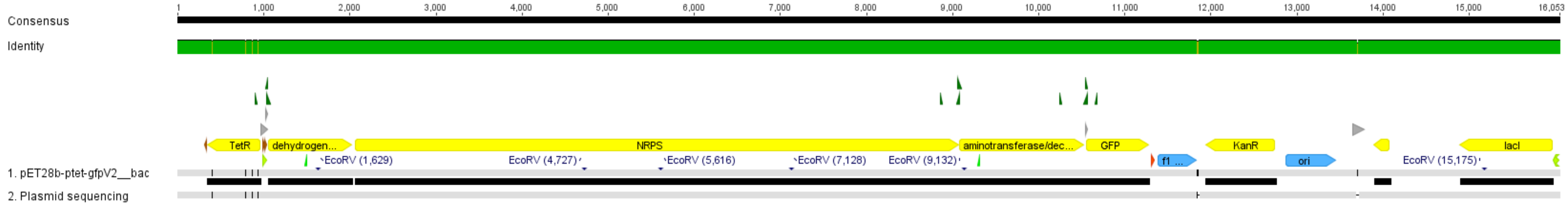

Figure S8. Schematic representation of the plasmid sequencing results of pET28b-ptetO::*bac*::*gfp*. The pairwise identity within the insert region is 100%.

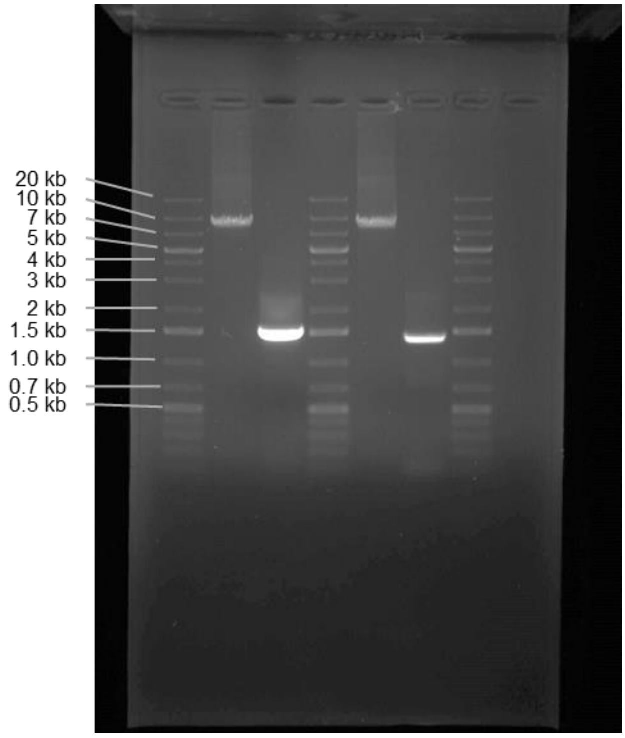

Figure S9: Uncropped gel image of Figure 2b.

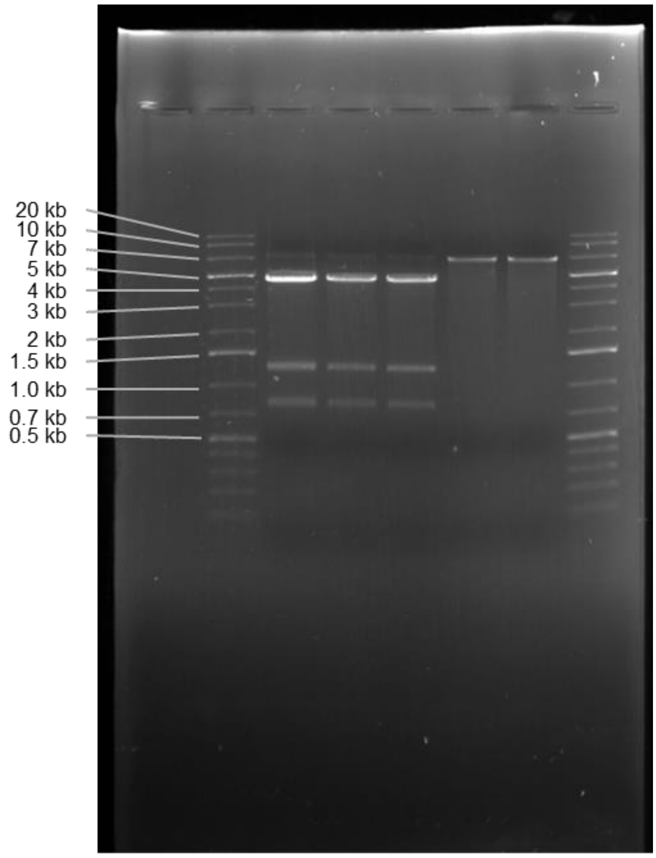

Figure S10: Uncropped gel image of Figure 2c.

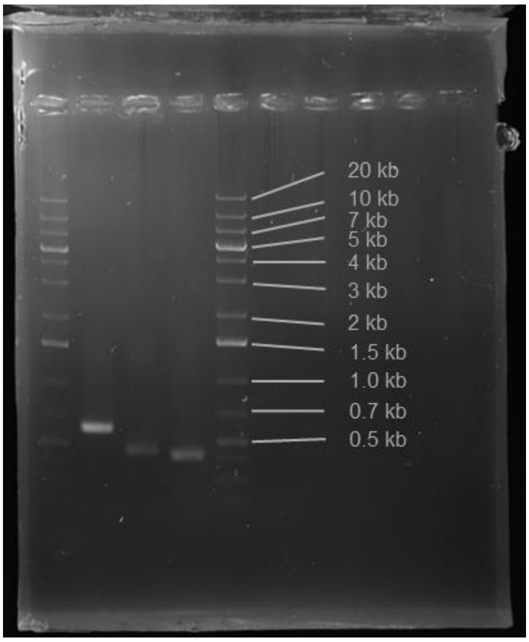

Figure S11: Uncropped gel image of Figure 2d.

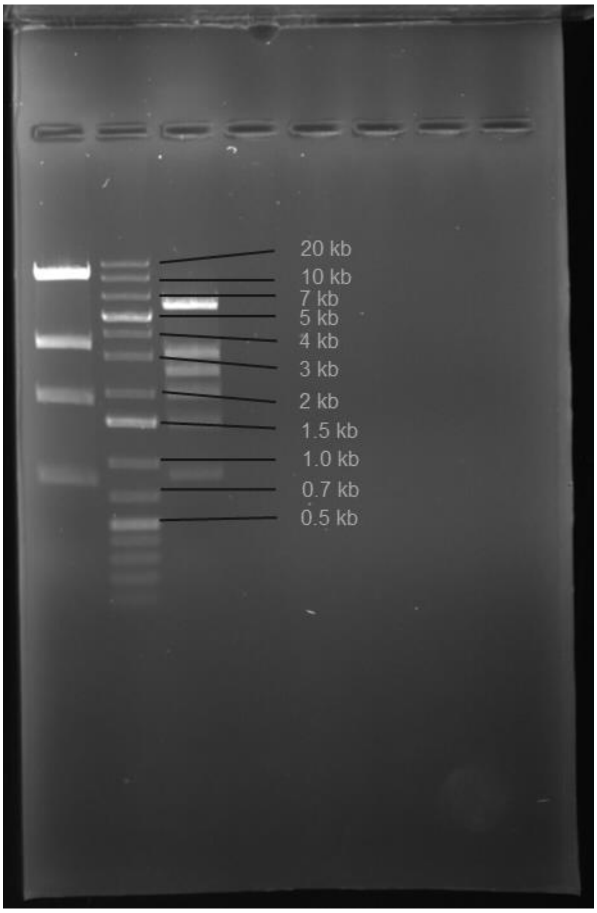

Figure S12: Uncropped gel image of Figure 2e.

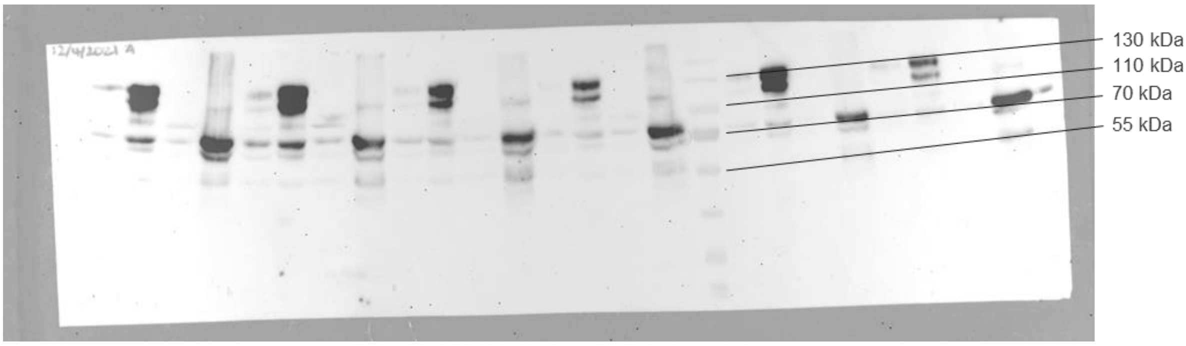

Figure S13: Uncropped image of integrin/lamin-blot from Figure S4.

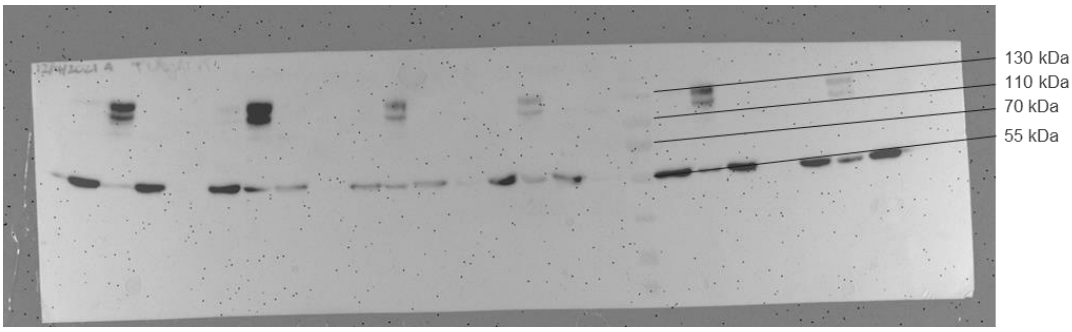

Figure S14: Uncropped image of tubulin-blot from Figure S4.

Supplementary Data

1. **Filename: Supplementary Data 1**

   Description: List of proGenomes v2 accession numbers used for Figure S7.

2. **Filename: Supplementary Data 2**

   Description: The source data behind the graphs in the paper.

NMR-Data

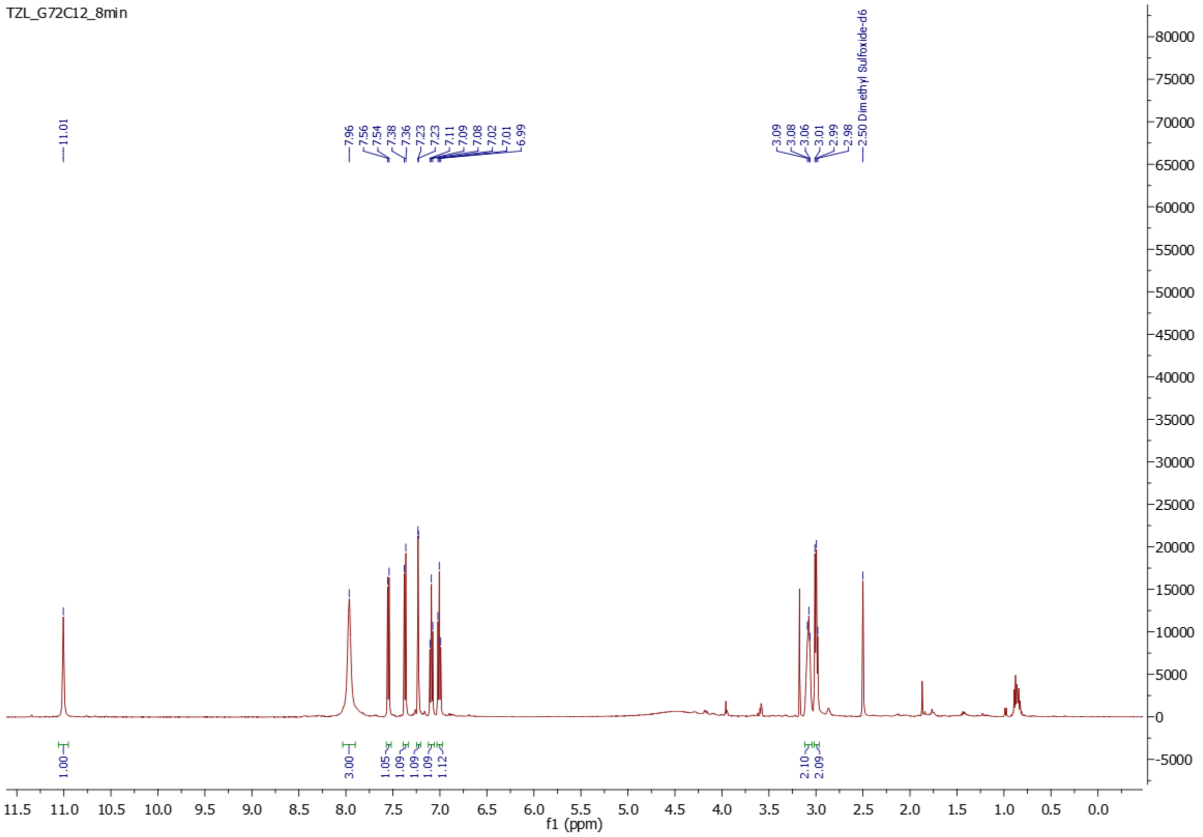

Figure S15. ^{1}H-NMR of tryptamine in DMSO-d6.

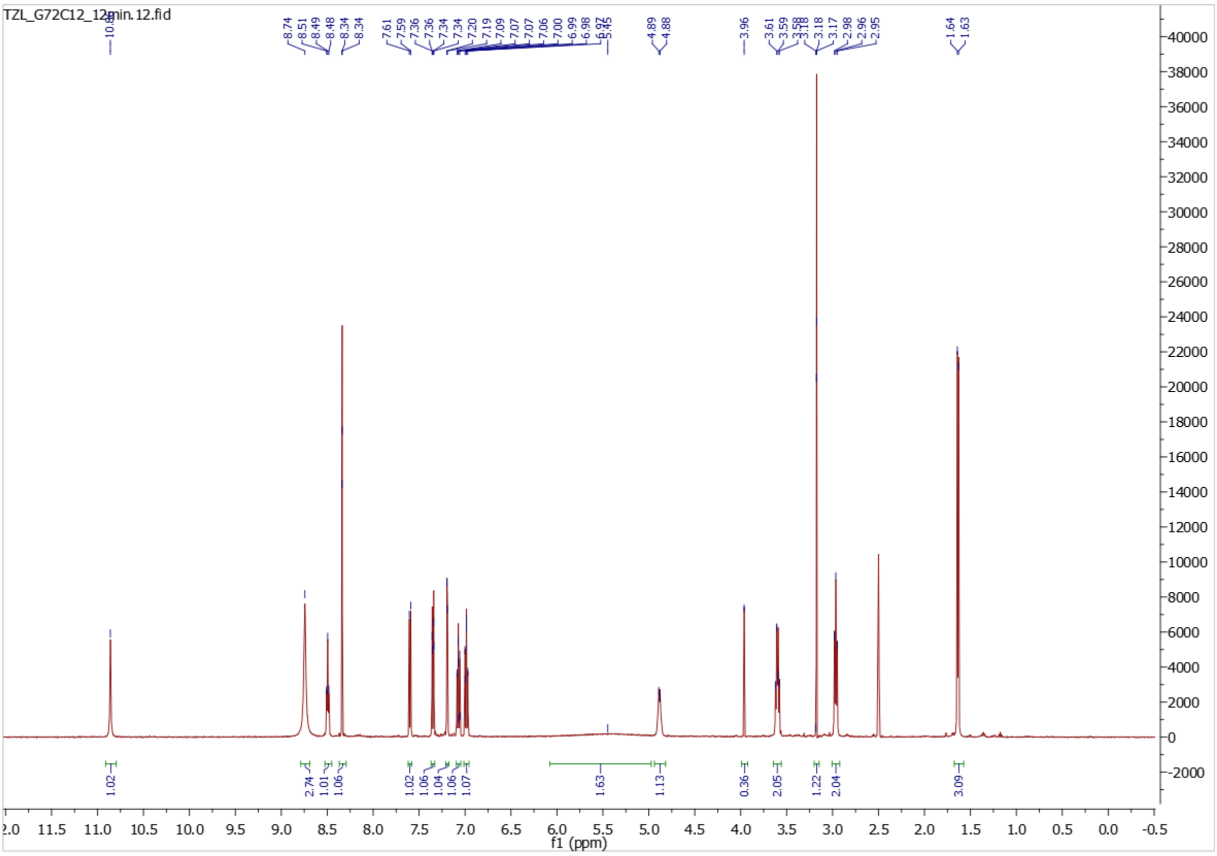

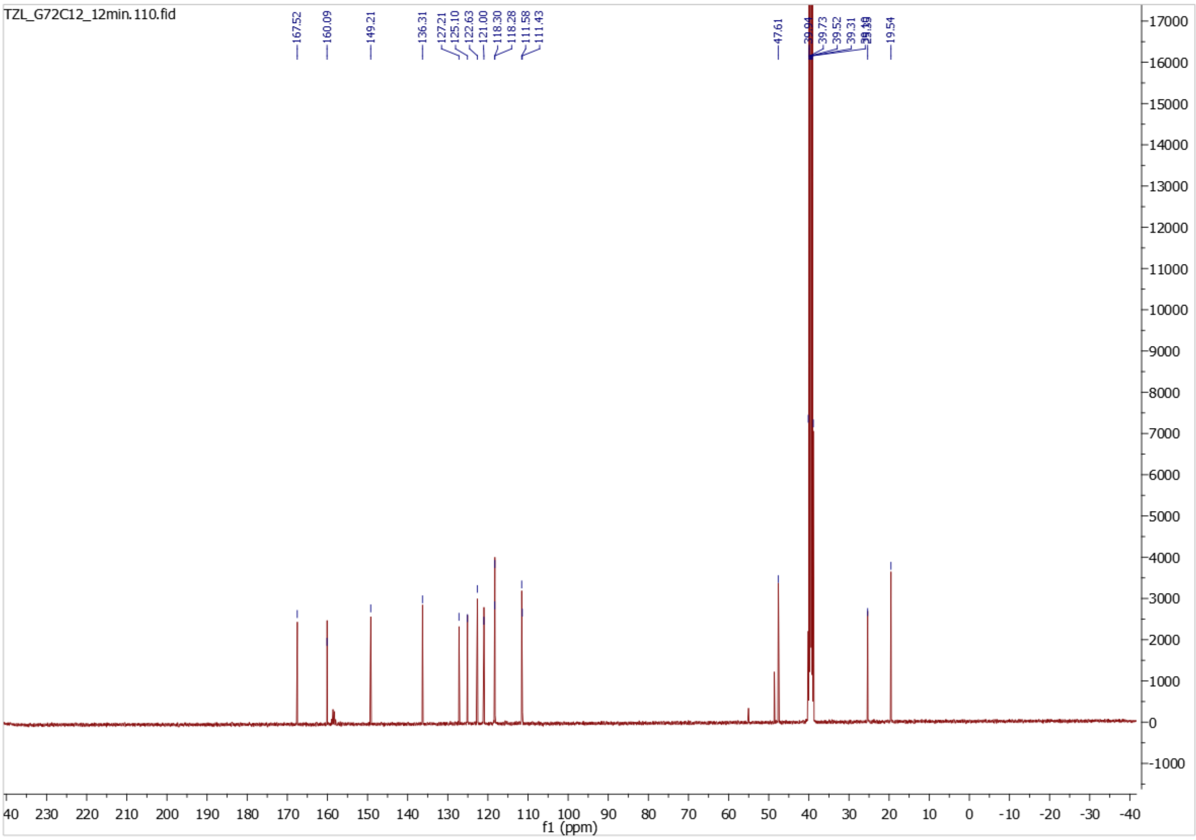

Figure S16. ^{1}H and ^{13}C NMR-spectra of bacillamide D in DMSO-d6.

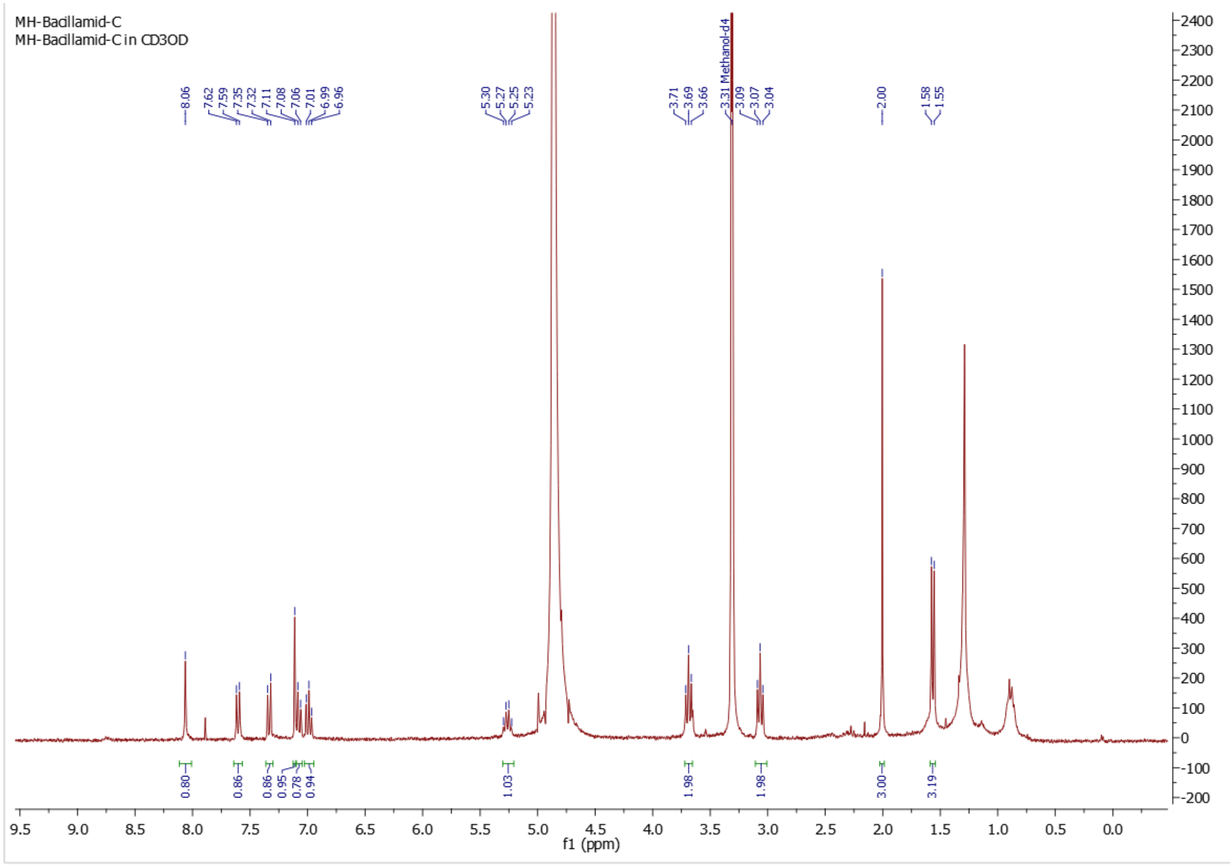

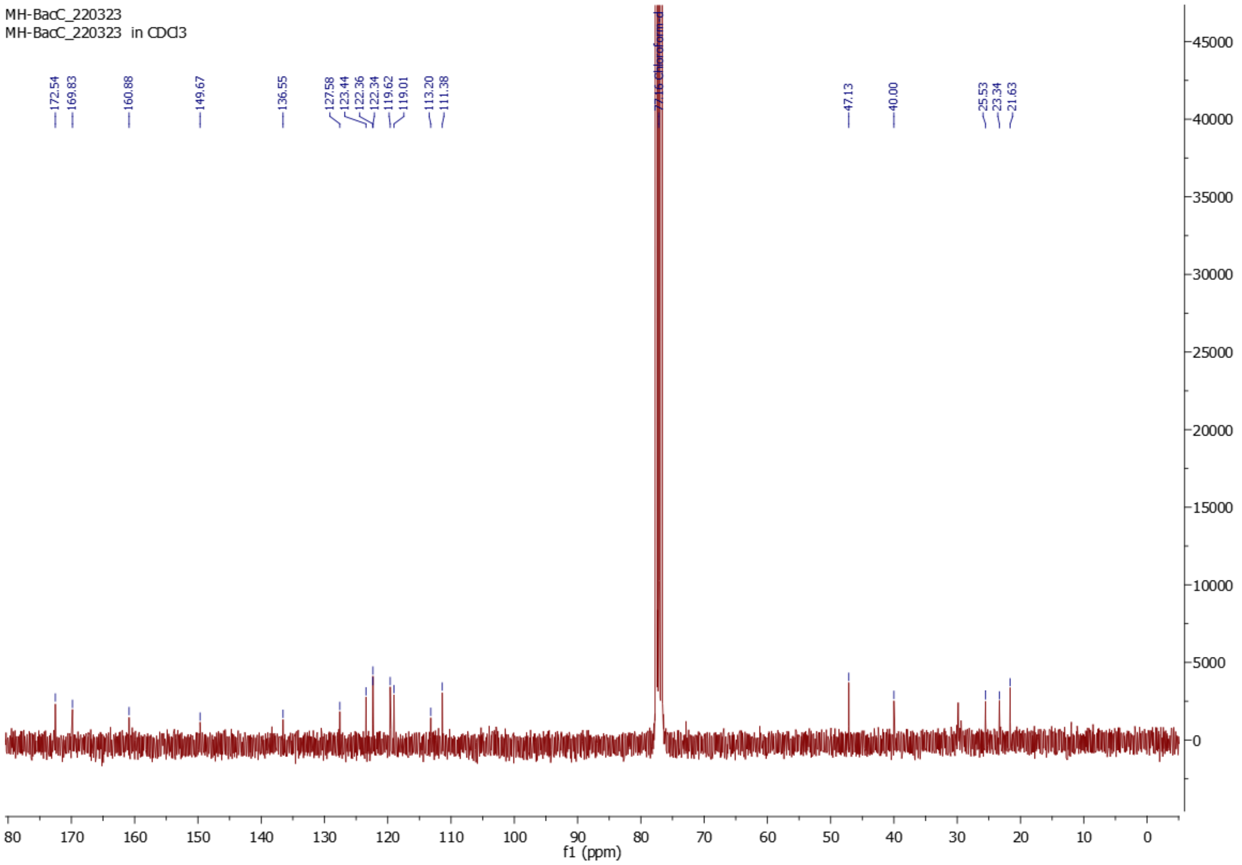

Figure S17. ^{1}H and ^{13}C NMR-spectra of bacillamide C in CD_3OD and $CDCl_3$, respectively.

Supplementary References

1. Pfeifer, B. A. & Khosla, C. Biosynthesis of polyketides in heterologous hosts. *Microbiol. Mol. Biol. Rev.* **65,** 106–118; 10.1128/MMBR.65.1.106–118.2001 (2001).

2. Lagkouvardos, I. *et al.* The Mouse Intestinal Bacterial Collection (miBC) provides host-specific insight into cultured diversity and functional potential of the gut microbiota. *Nat. Microbiol.* **1,** 16131; 10.1038/NMICROBIOL.2016.131 (2016).

3. Duell, E. R. *et al.* Direct pathway cloning of the sodorifen biosynthetic gene cluster and recombinant generation of its product in E. coli. *Microb. Cell Fact.* **18,** 32; 10.1186/s12934-019-1080-6 (2019).

4. Felchle, H. *et al.* Novel Tumor Organoid-Based Mouse Model to Study Image Guided Radiation Therapy of Rectal Cancer After Noninvasive and Precise Endoscopic Implantation. *Int. J. Radiat. Oncol. Biol. Phys.* **118,** 1094–1104; 10.1016/j.ijrobp.2023.10.008 (2024).

5. Mende, D. R. *et al.* proGenomes2: an improved database for accurate and consistent habitat, taxonomic and functional annotations of prokaryotic genomes. *Nucleic Acids Res.* **48,** D621-D625; 10.1093/nar/gkz1002 (2020).

6. Gurevich, A., Saveliev, V., Vyahhi, N. & Tesler, G. QUAST: quality assessment tool for genome assemblies. *Bioinformatics* **29,** 1072–1075; 10.1093/bioinformatics/btt086 (2013).

7. Parks, D. H., Imelfort, M., Skennerton, C. T., Hugenholtz, P. & Tyson, G. W. CheckM: assessing the quality of microbial genomes recovered from isolates, single cells, and metagenomes. *Genome Res.* **25,** 1043–1055; 10.1101/gr.186072.114 (2015).

8. Seemann, T. Prokka: rapid prokaryotic genome annotation. *Bioinformatics* **30,** 2068–2069; 10.1093/bioinformatics/btu153 (2014).

9. Tonkin-Hill, G. *et al.* Producing polished prokaryotic pangenomes with the Panaroo pipeline. *Genome Biol.* **21,** 180; 10.1186/s13059-020-02090-4 (2020).

10. Nguyen, L.-T., Schmidt, H. A., Haeseler, A. von & Minh, B. Q. IQ-TREE: a fast and effective stochastic algorithm for estimating maximum-likelihood phylogenies. *Mol. Biol. Evol.* **32,** 268–274; 10.1093/molbev/msu300 (2015).

11. Minh, B. Q., Nguyen, M. A. T. & Haeseler, A. von. Ultrafast approximation for phylogenetic bootstrap. *Mol. Biol. Evol.* **30,** 1188–1195; 10.1093/molbev/mst024 (2013).

12. Letunic, I. & Bork, P. Interactive Tree Of Life (iTOL) v5: an online tool for phylogenetic tree display and annotation. *Nucleic Acids Res.* **49,** W293-W296; 10.1093/nar/gkab301 (2021).

13. Carroll, L. M., Wiedmann, M. & Kovac, J. Proposal of a Taxonomic Nomenclature for the Bacillus cereus Group Which Reconciles Genomic Definitions of Bacterial Species with Clinical and Industrial Phenotypes. *mBio* **11**; 10.1128/mBio.00034-20 (2020).

14. Carroll, L. M., Cheng, R. A. & Kovac, J. No Assembly Required: Using BTyper3 to Assess the Congruency of a Proposed Taxonomic Framework for the Bacillus cereus Group With Historical Typing Methods. *Front. Microbiol.* **11,** 580691; 10.3389/fmicb.2020.580691 (2020).
