# Supplementary material for: Bacillamide D produced by Bacillus cereus from the mouse intestinal bacterial collection (miBC) is a potent cytotoxin in vitro
Source: Commun Biol. 2024 May 28;7:655. doi: 10.1038/s42003-024-06208-3 (PMC11133360; doi:10.1038/s42003-024-06208-3)
Supplement: Supplementary file 3 — Description of Additional Supplementary Materials [file 42003_2024_6208_MOESM3_ESM.docx]

**Description of Additional Supplementary Files**

**File name:** Supplementary Data 1

**Description:** List of proGenomes v2 accession numbers used for Figure S7

**File name:** Supplementary Data 2

**Description:** The source data behind the graphs in the paper.
